# Strategic resource allocation for malaria elimination in endemic settings: a systematic review of cost-effectiveness evidence

**DOI:** 10.3389/fpubh.2025.1718225

**Published:** 2026-02-09

**Authors:** Misra Helma Firdaus, Dina Syazana Ho Imran, Sharifa Ezat Wan Puteh, Tam Jenn Zhueng, Mohd Hafizi Abdul Hamid, Zulkarnain MD Idris, Mohd Rizal Abdul Manaf

**Affiliations:** 1 Department of Public Health Medicine, Faculty of Medicine, Universiti Kebangsaan Malaysia, Kuala Lumpur, Malaysia; 2 Vector Borne Disease, Disease Control Division, Ministry of Health (MOH), Putrajaya, Malaysia; 3 Department of Parasitology and Entomology, Faculty of Medicine, Universiti Kebangsaan Malaysia, Kuala Lumpur, Malaysia

**Keywords:** cost-effectiveness, economic evaluation, endemic settings, malaria, resource allocation, socio-ecological model

## Abstract

**Introduction:**

Malaria remains a significant global health burden, especially in endemic regions where efficient use of limited resources is critical. Economic evaluations provide essential evidence to guide strategic resource allocation and optimise intervention outcomes. This systematic review synthesises recent evidence (2018–2025) on the cost-effectiveness of malaria prevention and treatment interventions in endemic settings.

**Materials and methods:**

A systematic search of Scopus, Web of Science, and PubMed identified English-language studies published between 2018 and 2025. Eligible studies included full economic evaluations cost effectiveness (CEA), cost utility (CUA), and cost benefit analysis (CBA) assessing malaria prevention or treatment programs. Data extracted included intervention types, comparators, outcomes, costs, and incremental cost-effectiveness ratios (ICERs).

**Results:**

Eighteen studies met the inclusion criteria, mainly randomised trials evaluating insecticide-treated or long-lasting insecticidal nets (ITNs/LLINs), indoor residual spraying (IRS), diagnostics, treatment strategies, and combined interventions. Most were conducted in Sub-Saharan Africa LLINs and IRS were highly cost-effective, with costs per disability-adjusted life year (DALY) averted below national GDP thresholds. Combined approaches enhanced effectiveness in high-resistance or high-burden settings, while complex strategies, such as tafenoquine with G6PD screening, showed higher ICERs due to additional costs and operational challenges.

**Conclusion:**

Most malaria interventions in Sub-Saharan Africa were highly cost-effective, including LLINs (with PBO nets), IRS (Actellic/3GIRS), combined LLIN + IRS, SMC/PDMC, IPTp, and ACT + RDT. RTS,S vaccination and RDT + microscopy were moderately cost-effective, while G6PD screening with tafenoquine and traveller chemoprophylaxis were context-specific or less cost-effective. A socio-ecological framework underscores the need to align policies with economic evidence for optimal resource allocation and accelerated malaria elimination.

**Systematic review registration:**

https://www.crd.york.ac.uk/PROSPERO/view/CRD42024546911, PROSPERO Registration: CRD42024546911.

## 1 Introduction

Malaria remains a major global health challenge despite decades of control efforts. In 2023, an estimated 263 million cases were reported worldwide, corresponding to an incidence of 60.4 per 1,000 population at risk an increase of 11 million cases from 2022. The WHO African Region bears the heaviest burden, accounting for 94% of global cases. Malaria deaths reached 597,000 in 2023 (13.7 per 100,000 population), reflecting only modest improvement compared with 2020 (1). Pregnancy remains a particularly high-risk period: in 33 moderate-to-high transmission countries, 12.4 million pregnancies were infected, with intermittent preventive treatment in pregnancy (IPTp) averting approximately 551,000 cases of low birthweight (1–3).

The WHO Global Technical Strategy (GTS) for Malaria aims to reduce incidence and mortality by 40% by 2020, 75% by 2025, and 90% by 2030, using 2015 as a baseline (1). However, progress has been uneven: by 2023, only 38% of endemic settings achieved the 2020 mortality milestone, while 17% reported increased mortality (1, 4). In contrast, the WHO South-East Asia Region is on track to meet 2025 and 2030 milestones, illustrating that sustained elimination is achievable under the right conditions (1, 4, 5).

Despite strong global commitments, progress toward the GTS milestones has been slower than anticipated. According to the *World Malaria Report 2024*, multiple structural and operational challenges have contributed to these delays. Persistent funding shortages continue to impede the expansion and long-term sustainability of malaria interventions. At the same time, widespread insecticide resistance has reduced the effectiveness of traditional vector-control tools, particularly in high-transmission settings (1, 5).

The emergence and geographic spread of artemisinin partial resistance have further compromised the efficacy of first-line antimalarial therapies in several endemic regions. Additionally, health-system constraints, including workforce shortages, limited surveillance capacity, and recurrent supply-chain disruptions, continue to hinder the effective implementation of malaria control programmes (2, 3).

Vector control and preventive measures have been central to malaria reduction. In sub-Saharan Africa, household ownership of insecticide-treated nets increased from 68% in 2015 to 73% in 2023, while usage rose from 46 to 52% (6). Deployment of next-generation nets expanded rapidly, with pyrethroid-piperonyl butoxide (PBO) nets and dual active ingredient ITNs accounting for 58 and 20% of distributions, respectively, in 2023 (1, 6). Indoor residual spraying (IRS) was implemented in 42 endemic settings, but population-wide coverage remained limited (1.6%), though targeted coverage within intervention areas reached 88.4% (7). Seasonal malaria chemoprevention (SMC) also expanded to 19 countries, reaching 53 million children in 2023, with Nigeria alone accounting for more than half of all treatments (1, 7).

Despite these gains, malaria elimination efforts are constrained by insufficient resources. Global funding in 2023 amounted to USD 4.0 billion far short of the USD8.3 billion required leaving a gap of USD4.3 billion (1). Although domestic financing has grown to 37% of total contributions, most support still depends on international donors such as the Global Fund and bilateral partners. Research and development (R&D) showed modest recovery, with USD 690 million invested in 2023, yet critical gaps persist, particularly in biologics and next-generation tools. Without major increases, per capita funding is projected to meet only 60% of requirements by 2030 (1, 5, 8).

Under such constraints, prioritising cost-effective interventions is essential to maximise health gains. Economic evaluations provide crucial evidence for policymakers, health systems, and communities to allocate resources strategically. The social ecological model (SEM) offers a useful framework for situating malaria interventions across multiple levels: individual strategies such as chemoprophylaxis; interpersonal and family-based approaches such as IPTp; community-level strategies including integrated community case management (iCCM) and mass drug administration; and system- and policy-level strategies such as IRS, long-lasting insecticidal nets (LLINs), seasonal chemoprevention, and malaria vaccination programmes (9, 10).

This systematic review synthesises recent evidence on the cost-effectiveness of malaria control and elimination strategies. This review is critical for guiding strategic resource allocation, supporting policy and programme prioritisation, and accelerating progress toward the 2030 elimination targets (1, 11). Despite several systematic reviews and economic evaluations of malaria interventions, none have integrated updated evidence on cost-effectiveness, strategic resource allocation, and socio-ecological modelling across multiple endemic settings. To highlight this distinction, the summary of similar PROSPERO and Cochrane records, illustrating how our review differs in terms of publication year, objectives, outcomes, and methodological framework (Supplementary Table 1).

## 2 Materials and methods

### 2.1 Research question formulation

This review aims to explore the research question “*Which malaria interventions are most cost-effective for reducing disease burden in endemic settings?”* It synthesises cost-effectiveness evidence across the full spectrum of malaria interventions, from individual to policy levels. The review does not seek to directly compare, for example, individual-level strategies with policy-level strategies, but rather to identify which interventions within and across these levels represent the most efficient use of resources.

The review employs the PICO framework (Population, Intervention, Comparator, Outcome) to formulate the research question, ensuring a comprehensive and systematic synthesis of the available evidence.

- *Population (P)*: individuals, households, and communities living in malaria-endemic regions, particularly in low- and middle-income countries.
- *Intervention (I)*: malaria prevention and control strategies, including long-lasting insecticidal nets (LLINs)/insecticide-treated nets (ITNs), indoor residual spraying (IRS), outdoor residual spraying (ORS), larviciding, mass drug administration (MDA), diagnostic tools, and treatment approaches.
- *Comparator (C)*: the comparators typically comprised are usual care or standard practice, alternative interventions, or “Do-nothing” or placebo scenarios, were assessed against a baseline without a specific intervention.
- *Outcome (O)*: economic evaluation measures such as ICERs, cost per DALY averted, cost per case, or cost per death averted.

Using PICO allowed systematic integration of costs, health outcomes, and intervention effectiveness, providing a robust framework for evaluating cost-effectiveness and informing resource allocation in malaria elimination programmes.

### 2.2 Data source and search strategy

Data for this review were sourced using three major electronic databases: Scopus, PubMed, and Web of Science (WOS). These databases were selected for their extensive coverage of peer-reviewed publications in global health, economics, and malaria research.

The search was restricted to studies published from 2018 to 2025 to capture the most recent evidence on malaria elimination interventions and their economic evaluations. This timeframe was chosen to reflect current practices, methodologies, and policy-relevant findings in the field of malaria cost-effectiveness.

The search strategy employed combinations of keywords and Boolean operators related to malaria, economic evaluation, cost-effectiveness, and intervention strategies. Search terms were structured according to the PICO (Population, Intervention, Comparator, Outcome) framework to ensure systematic and comprehensive retrieval of relevant studies. The detailed search terms and strategies applied in each database are provided in Supplementary Table 2.

A total of 442 articles were retrieved across the three databases. Only 18 studies met the eligibility requirements and were included in this review. The study selection process is presented in the PRISMA 2020 flow diagram.

Most included studies were conducted in Sub-Saharan Africa, reflecting the region’s high malaria burden and concentration of intervention research, while additional studies came from Southeast and South Asia, consistent with the global distribution of malaria endemicity. By focusing on empirical economic evaluations, this review provides a robust evidence base to guide strategic resource allocation for malaria interventions.

The study protocol was registered with PROSPERO under the registration number CRD42024546911, available at: https://www.crd.york.ac.uk/PROSPERO/view/CRD42024546911.

### 2.3 Inclusion and exclusion criteria

The inclusion and exclusion criteria were carefully formulated to ensure the relevance, quality, and methodological rigour of the studies reviewed. Inclusion criteria were defined to capture original empirical research that directly assessed malaria elimination interventions in endemic settings through an economic evaluation framework.

Eligible studies included cost-effectiveness, cost-utility, or cost-benefit analyses that evaluated interventions such as long-lasting insecticidal nets (LLINs), indoor residual spraying (IRS), outdoor residual spraying (ORS), mass drug administration (MDA), larval source management (LSM), or combined strategies. Studies were included if they reported measurable economic outcomes, such as incremental cost-effectiveness ratios (ICER), cost per disability-adjusted life year (DALY) averted, cost per case averted, or cost per death averted.

Exclusion criteria were applied to maintain the focus and integrity of the review. Review papers, editorials, commentaries, conference abstracts, and grey literature were excluded, as they do not provide original data suitable for systematic synthesis. Studies were also excluded if they lacked economic evaluation components, did not report relevant outcome measures, or were conducted outside malaria-endemic settings.

By prioritising studies that combine empirical data with economic evaluation methods, this review provides a robust evidence base to inform strategic resource allocation decisions and guide policymakers on the most cost-effective malaria interventions for reducing disease burden in endemic regions.

### 2.4 Data extraction and synthesis

All authors (MHF, MRAM, SEWP, DSHI, TJZ, MHAH, ZMI) independently extracted information from each eligible article using a standardised Excel spreadsheet. The extraction process was reviewed collaboratively, with discrepancies resolved through discussion and consensus among the review team. This ensured consistency, accuracy, and methodological rigour throughout the process.

Data extraction was guided by a structured form designed to capture the key characteristics of each study, including authorship, year of publication, country or region, study design, population characteristics, type of malaria intervention (e.g., LLIN, IRS, ORS, MDA, LSM), and the outcomes assessed. Outcomes were categorised into two broad domains. Economic outcomes (e.g., incremental cost-effectiveness ratio (ICER), cost per disability-adjusted life year (DALY) averted, cost per case averted, cost per death averted) and public health impact (e.g., malaria incidence reduction, mortality reduction).

Data from the included studies was extracted using a standardised form, capturing study characteristics, intervention details, outcomes, and cost-effectiveness results. The strength of cost-effectiveness evidence was assessed using WHO-CHOICE thresholds and classified into five categories: Strong, Moderate, Limited, Emerging, and Context-Specific based on consistency, robustness, and contextual generalizability (5). Specifically:

- *Strong evidence* (●●●): interventions consistently demonstrated highly cost-effective results (ICER <1× GDP per capita) across multiple robust studies and varied endemic settings.
- *Moderate evidence* (●●○): interventions were cost-effective (ICER 1–3× GDP per capita) but showed some variability across contexts or study designs.
- *Limited evidence* (●○○): evidence was mixed or uncertain due to small sample sizes, methodological limitations, or context-specific factors.
- *Emerging evidence* (◇): novel interventions with promising early results, but limited replication or comparative data.
- *Context-specific evidence* (◆): cost-effectiveness dependent on unique local or epidemiological conditions, limiting generalisability.

This framework provides a transparent, reproducible approach to interpret cost-effectiveness evidence beyond ICER values, supporting informed policy recommendations. Following data extraction, a narrative synthesis was conducted to compare and integrate the cost-effectiveness evidence across interventions and geographic settings.

This structured approach allowed the review to identify patterns in economic efficiency, highlight which interventions provide the best value for money in endemic regions, and assess their implications for strategic resource allocation in malaria elimination and elimination programmes.

#### 2.4.1 Eligibility

The review selection process is illustrated in Supplementary Figure 1 using a PRISMA 2020 flow diagram. A total of 1,224 records were initially identified through comprehensive searches across three databases: Scopus, PubMed, and Web of Science. After removing 499 duplicate records, 725 studies remained for title and abstract screening.

This screening was independently conducted by two reviewers (MHF, DSHI, SEWP) using predefined inclusion and exclusion criteria. At this stage, 678 records were excluded for reasons such as irrelevance to malaria elimination programmes, a focus on non-human populations (e.g., animal studies), methodological misalignment with public health or economic evaluation, insufficient reporting, and duplicates not previously removed. This process left 47 articles for full-text eligibility assessment.

The same two reviewers then evaluated the full-text articles to confirm their eligibility, with any disagreements resolved through consultation with a third reviewer (MRAM) to ensure consensus. Following this rigorous process, *18 studies* fully met the inclusion criteria and were included in the final synthesis.

The PRISMA flow diagram shown in Supplementary Figure 1 was incorporated as part of the quality appraisal process, providing a transparent account of study selection and documenting the reasons for exclusion at each stage in accordance with the PRISMA 2020 guidelines.

### 2.5 Quality tool assessment

The appraisal was conducted independently by multiple reviewers (MRAM, SEWP, MHF, and DSHI), with discrepancies resolved through consensus to minimise bias and ensure transparency. Each study was assessed using the Joanna Briggs Institute (JBI) Critical Appraisal Tools, which evaluate domains such as methodological rigour, clarity of reporting, appropriateness of analysis, and potential sources of bias (12, 13).

Overall, the methodological quality of the included studies was high. Both cross-sectional studies achieved a perfect score (100%), demonstrating strong design and analytical robustness. Among the 16 randomised controlled trials (RCTs), most scored 92%, reflecting solid performance across sequence generation, blinding, allocation concealment, and validity of outcome measures, though minor limitations were noted in relation to blinding procedures. Importantly, no study was excluded based on quality appraisal. These findings indicate that the body of evidence synthesised in this review is methodologically robust and provides a reliable foundation for evaluating the cost-effectiveness of malaria interventions in Supplementary Table 3.

## 3 Result

### 3.1 The characteristics of the studies

This review included 18 studies from a variety of geographical settings. The majority of the studies, 13 in total, were conducted in Sub-Saharan Africa, which has a high malaria burden. Other studies came from Asia are China and India, South America (Brazil), and Europe (Paris). This distribution shows that the majority of cost-effectiveness evidence has been generated in high-burden, resource-limited settings, particularly within Sub-Saharan Africa, while fewer studies come from non-endemic or low-transmission areas.

The included studies were published between 2018 and 2025, indicating a relatively new evidence base. The first included study was published in 2018 (14), and the most recent appeared in 2024 (15). This reflects a growing body of literature on the economic evaluation of malaria interventions over the last 6 years, which is consistent with global policy emphasis on cost-effective resource allocation.

Supplementary Table 4 shows that the populations targeted differed between studies. Eight studies evaluated general populations, while others examined specific subgroups such as children (six studies), pregnant women (two studies), and travellers (one study). This variation suggests that both universal and high-risk groups have been prioritised in cost-effectiveness analyses, with children and pregnant women frequently mentioned due to their increased vulnerability to malaria in Sub-Saharan Africa.

### 3.2 Types of malaria interventions and economic evaluations

The interventions examined can be broadly classified into preventive, treatment, and diagnostic measures as shown in Supplementary Table 4:

- *Preventive measures (10 studies)*: these included combined long-lasting insecticidal nets (LLINs) and indoor residual spraying (IRS), LLINs with synergists (PBO), seasonal malaria chemoprevention (SMC), post-discharge malaria chemoprevention (PDMC), chemoprophylaxis in travellers, reactive focal mass drug administration (rfMDA), and intermittent preventive treatment (IPT) for pregnant women. Outcomes evaluated included averted cases, deaths, DALYs, and QALY gains.
- *Treatment measures* were evaluated in four studies: malaria vaccination (RTS,S/AS01), artemisinin-based combination therapy (ACT) combined with diagnostic testing, and integrated community case management (ICCM) with drug seller training. These studies measured cases averted, deaths averted, DALYs averted, and QALYs gained, with vaccination demonstrating high cost-effectiveness.
- *Diagnostic measures* in four studies: The focus was on rapid diagnostic testing (RDT) with microscopy, G6PD screening, and tafenoquine with prior G6PD testing. The primary outcomes were cases avoided, DALYs avoided, and hospitalisations avoided, which are especially important for ensuring the safe use of primaquine and tafenoquine in vivax malaria control.

These 18 studies collectively assessed diverse malaria control strategies across preventive, treatment, and diagnostic domains. Economic evaluations consistently reported cases, deaths, and DALYs averted as primary outcomes, with some also measuring QALYs and hospitalisation avoidance. The following section synthesises these findings to highlight comparative cost-effectiveness patterns and policy relevance.

### 3.3 The synthesis of cost-effectiveness evidence and policy implications

To provide a concise and actionable overview for policymakers, the cost-effectiveness findings from the 18 included studies were synthesised and consolidated into Supplementary Table 5. Interventions were grouped into three primary categories: Preventive, Treatment, and Diagnostics. The synthesis integrates comparative information on the number of supporting studies, incremental cost-effectiveness ratios (ICERs), dominance status, cost-effectiveness classification, and resulting policy recommendations.

Overall, the evidence demonstrates a strong economic justification for sustaining and scaling several core malaria interventions. Among vector control strategies, both indoor residual spraying (IRS)—particularly with long-lasting formulations such as *Actellic 300CS* and *3GIRS*, and long-lasting insecticidal nets (LLINs), including PBO-enhanced nets, were consistently reported as highly cost-effective, with ICERs well below regional GDP per capita thresholds. The combined LLINs + IRS strategy yielded the greatest impact in areas of high transmission and insecticide resistance, underscoring its value as a priority intervention package.

For chemoprevention, seasonal malaria chemoprevention (SMC) and post-discharge malaria chemoprevention (PDMC) demonstrated strong economic performance, often being dominant (more effective and less costly than alternatives). These interventions should remain top priorities for children under five in seasonal transmission zones.

Under the treatment category, both intermittent preventive treatment in pregnancy (IPTp) and the use of artemisinin-based combination therapy (ACT) guided by rapid diagnostic testing (RDT) were frequently dominant or highly cost-effective. Their integration into routine antenatal care and standard case management is essential to sustain maternal and child survival gains and ensure rational antimalarial use. The RTS,S malaria vaccine also showed cost-effective to highly cost-effective results, supporting its role as a complementary tool where financing and delivery infrastructure permit.

In contrast, the Diagnostics group exhibited context-dependent cost-effectiveness. Combined RDT and microscopy remained broadly cost-effective and should continue as part of diagnostic-led strategies to reduce overtreatment. However, G6PD screening prior to tafenoquine use for *P. vivax* elimination, while clinically indispensable, was not dominant, and its cost-effectiveness varied considerably depending on screening costs, indicating the need for careful piloting before wide implementation.

Similarly, chemoprophylaxis for travellers, though cost-effective in non-endemic contexts, is not a priority intervention for endemic regions. This synthesis provides an integrated framework for evidence-informed resource allocation, allowing decision-makers to identify high-value interventions for scale-up while recognising context-specific tools requiring further evaluation or targeted deployment.

Overall, the evidence indicates that most malaria interventions in Sub-Saharan Africa are highly cost-effective. Preventive strategies, including LLINs (with PBO nets), IRS (Actellic/3GIRS), and combined LLIN + IRS, showed the greatest impact. Chemoprevention (SMC/PDMC) and treatment interventions (IPTp, ACT + RDT) were also highly cost-effective, whereas diagnostics and emerging interventions, such as G6PD screening with tafenoquine and traveller chemoprophylaxis, were context-specific or moderately cost-effective. These findings provide actionable guidance for prioritising resource allocation in endemic settings.

### 3.4 Evidence strength and policy implications of malaria interventions

The strength of cost-effectiveness evidence was assessed using WHO-CHOICE thresholds based on GDP per capita criteria and classified as Strong, Moderate, Limited, Emerging, and Context-Specific, considering consistency, robustness, and contextual generalisability (1, 5). Overall, most interventions demonstrated moderate to strong cost-effectiveness evidence, indicating a robust economic basis for continued investment.

Approximately 28% (5/18) of studies demonstrated strong evidence (●●●), particularly for integrated LLIN plus IRS, IRS rotations with Actellic 300 CS or 3GIRS products, and malaria vaccination (RTS,S/AS01). These consistently high-quality findings provide the clearest justification for sustained and prioritised resource allocation in endemic, high-burden areas.

A larger proportion, around 39% (7/18), provided moderate evidence (●●○), covering interventions such as seasonal malaria chemoprevention (SMC), LLIN with PBO synergists, intermittent preventive treatment in pregnancy, ACT combined with RDT, and reactive focal mass drug administration. These interventions remain cost-effective in specific contexts, suggesting that resources should be strategically allocated based on epidemiological setting and health system capacity.

By contrast, 17% (3/18) of studies offered limited evidence (●○○), including delivery modes for LLIN, ICCM training programmes, and RDT plus microscopy. These may have programmatic benefits but appear less cost-effective compared to preventive and treatment-focused strategies, thus warranting cautious allocation.

Emerging and niche interventions accounted for a smaller share of the evidence base: 11% (2/18) were classified as emerging (◇)—notably chemoprophylaxis in travellers and G6PD screening, while 6% (1/18) was context-specific (◆), represented by tafenoquine treatment following G6PD screening for *P. vivax*. These findings highlight promising but still limited or context-dependent options, requiring further research and cost analyses before large-scale adoption.

Overall, among the 18 studies in Supplementary Figure 2, the majority demonstrated moderate (38.9%) or strong (27.8%) evidence strength, suggesting that most malaria interventions evaluated are supported by relatively robust cost-effectiveness findings. Limited (16.7%), emerging (11.1%), and context-specific (5.6%) evidence categories were less common, highlighting areas where additional research and policy evaluation are required.

## 4 Discussion

### 4.1 Evidence strength and policy implications

This review demonstrates that cost-effectiveness evidence for malaria interventions is not uniform but varies in strength and applicability, with important implications for programme planning and policy. Interventions consistently supported by strong evidence, such as integrated LLIN plus IRS, IRS rotations with Actellic 300 CS or 3GIRS products, and the RTS,S/AS01 vaccine, offer the clearest justification for sustained investment. Even under constrained budgets, these strategies represent high-value interventions and should remain priorities for countries aiming to maximise health and economic returns (16–18).

The economic synthesis presented in the enhanced summary (Supplementary Table 5) directly substantiates these conclusions. It shows that these interventions not only have strong evidence but also consistently outperform GDP-based cost-effectiveness thresholds, being classified as *highly cost-effective* or *cost-effective*. Furthermore, the provision of cost ranges (in USD) offers policymakers a concrete estimate of the financial investment required, filling a critical gap often present in meta-analyses of economic evaluations. This dual lens of cost and cost-effectiveness reinforces their economic justification and underscores their strategic value in resource allocation (1, 5).

Interventions with moderate evidence, including SMC, IPTp, PBO-LLINs, ACT plus RDT, and rfMDA, underscore the need for contextual adaptation. Their cost-effectiveness is shaped by factors such as epidemiological setting, resistance profiles, and delivery capacity. Rather than universal application, policymakers should deploy these strategies selectively, targeting areas where effectiveness is likely to be maximised. Such tailored deployment improves value for money while accommodating heterogeneity in outcomes across different settings (2, 3, 7, 17).

Interventions with limited or emerging evidence, including ICCM, RDT plus microscopy, chemoprophylaxis for high-risk travellers, and tafenoquine following G6PD screening, require cautious consideration. While they may offer operational or clinical benefits, their broader economic justification remains either weak or highly context-dependent often failing to meet standard cost-effectiveness thresholds. These patterns illustrate that not all innovations translate into economic efficiency, and further empirical validation is required before large-scale investment or policy endorsement (19–21).

### 4.2 Research gaps and future priorities

Critical gaps remain in the evidence base for novel and context-specific interventions. In particular, economic evaluations for zoonotic malaria and *Plasmodium vivax* remain scarce, despite their growing significance in Southeast Asia and other regions where elimination goals are under threat (1, 22–24). Addressing these gaps through real-world implementation studies, modelling analyses, and long-term cost-effectiveness evaluations is essential for guiding future programme strategies.

### 4.3 Policy and strategic resource allocation: a socioecological perspective

The socioecological model (SEM) provides a useful framework for situating malaria interventions within multiple, interacting levels, as shown in Supplementary Figure 3 (10, 11). It supports strategic interpretation of findings but was not used to define comparators within the cost-effectiveness analysis. Individual level: Chemoprophylaxis for high-risk travellers, tafenoquine post-G6PD screening, and post-discharge malaria chemoprevention (PDMC) represent niche, context-specific interventions.

At this level, the effectiveness of malaria elimination measures such as insecticide-treated nets (ITNs) and indoor residual spraying (IRS) can be directly enhanced by behavior change campaigns and culturally sensitive education programmes (14, 21–27). These interventions improve knowledge, correct misconceptions, and promote preventive behaviors. Effectiveness can be tracked through indicators such as ITN adoption, IRS participation, and proper usage rates. Studies show that culturally tailored education significantly improves ITN compliance (28, 29). Advances such as drones for larval source management and genetic modification of mosquitoes are also emerging at this level (12, 13).

At family level, intermittent preventive treatment in pregnancy (IPTp) with dihydroartemisinin–piperaquine and seasonal malaria chemoprevention (SMC) for children under five demonstrate moderate cost-effectiveness (1, 7, 8). Mothers, as primary carers, shape ITN and IRS adherence, treatment-seeking, and household health decisions. Studies highlight that family health education and financial incentives increase ITN and IRS uptake (14, 22, 25–27). Measuring household-level malaria incidence and ITN use provides key insights into effectiveness (8, 30, 31).

Community level were IRS with Actellic 300 CS or 3GIRS products, and phased rollout of RTS,S/AS01, show strong cost-effectiveness (3, 7, 32, 33). Sustained budgetary allocations are justified, but effectiveness relies on health system capacity, surveillance, and workforce readiness. Examples include Uganda’s decentralised surveillance, which enables rapid outbreak response, and Rwanda’s mHealth reporting system, which improves data quality (34).

While, organisational/health system level such as IRS using Actellic 300 CS or 3GIRS products, alongside the phased rollout of RTS,S/AS01, demonstrates strong cost-effectiveness and justifies sustained budgetary allocations through health system mechanisms (16, 35).

Policy/national level: integrated LLIN plus IRS and universal LLIN coverage remain the most cost-effective strategies with strong evidence. Policies must be tailored to socio-cultural and epidemiological contexts (14, 27). High-transmission regions (e.g., Sub-Saharan Africa) require robust surveillance and integration of community health workers. Low-transmission settings (e.g., India, Brazil) demonstrate success with integrated strategies combining vector control, case management, and education (9, 36).

Southeast Asia has shown innovative models: Cambodia’s Malaria Elimination Action Framework combined malaria-dengue prevention, while Vietnam’s Village Health Collaboratives used local volunteers to build trust and track outcomes (9, 36). Digital health innovations (e.g., Rwanda’s mobile reporting) and cross-sector collaboration further enhance impact. Strengthening policies for sustainable funding, cultural adaptation, and community ownership is essential for long-term malaria elimination (1, 34).

When demographic variables are considered, the analysis becomes even more nuanced. The included studies covered a range of endemic settings, where intervention effectiveness was shaped by transmission intensity and health system readiness. Variability was also observed across types of malaria interventions and their health outcomes, particularly in malaria prevention outcomes such as reductions in incidence, prevalence, morbidity, and mortality.

Evidence consistently showed that high-burden countries with sustained LLIN and IRS deployment achieved the largest reductions in malaria incidence, while targeted strategies such as SMC and IPTp significantly lowered morbidity and mortality among vulnerable groups, including children under five and pregnant women (15, 37–40).

Taken together, these demographic dimensions reinforce that tailoring interventions to epidemiological settings, transmission patterns, and population vulnerability is essential to achieve both cost-effectiveness and maximal public health impact. Ultimately, integrating the SEM perspective with demographic realities offers a stronger, evidence-informed basis for strategic resource allocation in malaria-endemic settings (41–43).

## 5 Strengths and limitations

This review’s strengths include its broad scope across multiple countries and recent timeframe (2018–2025), providing a contemporary synthesis of malaria cost-effectiveness evidence. The integration of evidence strength assessment, both tabulated and visually represented, offers an additional dimension that enhances policy relevance. However, several limitations are acknowledged. First, substantial clinical and methodological heterogeneity precluded formal statistical assessment of publication bias and quantitative meta-analysis, necessitating a narrative synthesis, which is the recommended approach for such heterogeneous evidence.

Second, limiting the review to English-language publications may have introduced language bias. Finally, although the systematic review protocol was registered with PROSPERO, a major amendment was made during the review process; nonetheless, all methods were applied consistently in accordance with the registered protocol. Despite these limitations, this review contributes to bridging the gap between economic evaluation and policy translation, offering a structured framework to inform decision-making and optimise resource allocation.

## 6 Conclusion

This review underscores that most malaria interventions in Sub-Saharan Africa are supported by strong or moderate evidence of cost-effectiveness, particularly indoor residual spraying (IRS), long-lasting insecticidal nets (LLINs), and integrated preventive strategies, which consistently outperform GDP-based thresholds. Combining LLINs and IRS further enhances effectiveness, especially in high-transmission or drug-resistant areas. Emerging interventions, such as malaria vaccination or tafenoquine with G6PD screening, offer potential benefits but are constrained by high costs and logistical challenges. Framing malaria interventions within a socio-ecological model emphasises the need to align national policies with economic evidence, ensuring optimal allocation of resources from policy to community and individual levels, thereby accelerating progress toward malaria elimination across diverse Sub-Saharan African settings.

## Data Availability

The original contributions presented in the study are included in the article/Supplementary material, further inquiries can be directed to the corresponding author.
