# Supplementary material for: Strategic resource allocation for malaria elimination in endemic settings: a systematic review of cost-effectiveness evidence
Source: Front Public Health. 2026 Feb 9;13:1718225. doi: 10.3389/fpubh.2025.1718225 (PMC12926903; doi:10.3389/fpubh.2025.1718225)
Supplement: Supplementary file 1 [file Data_Sheet_1.pdf]

Supplementary Material

1 Supplementary Figures

1.1 Supplementary Figures

1.1.1 Supplementary Figure 1. Study selection process according to PRISMA flowchart

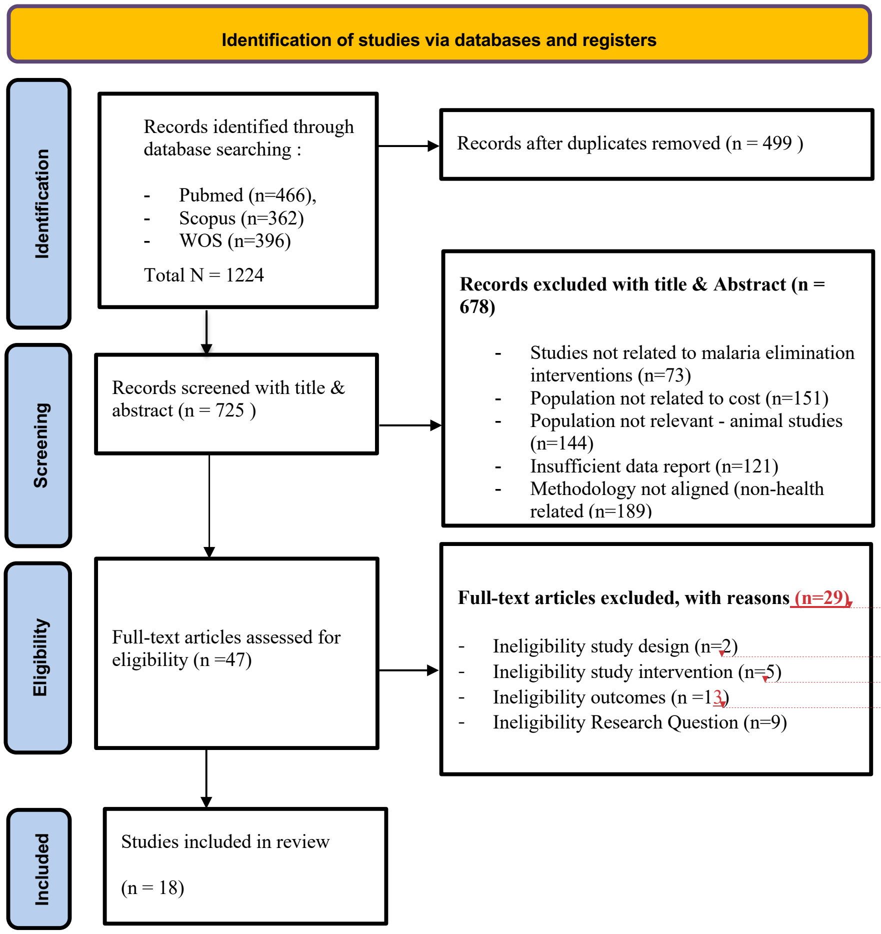

Deleted: :

Deleted: 1

Deleted: 1

Deleted: 1

1.1.2 Supplementary Figure 2. Distribution of evidence strength across malaria interventions (n = 18 studies).

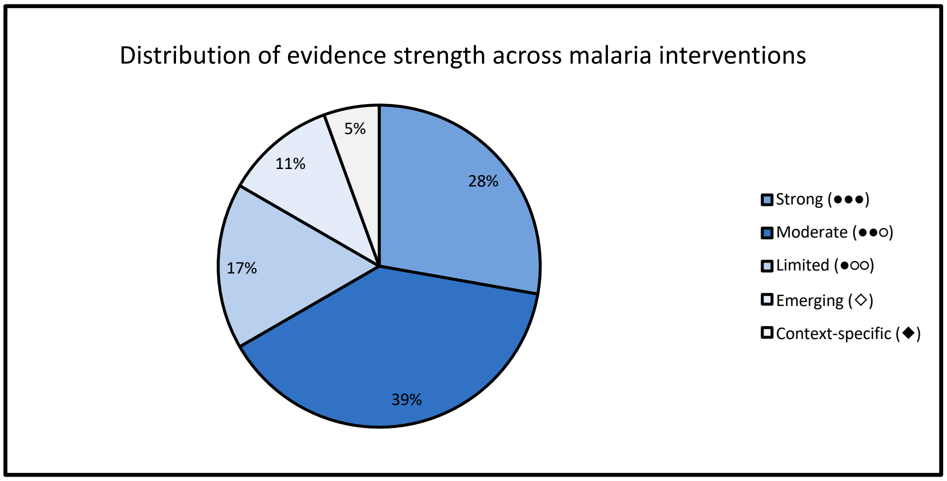

1.1.3 Supplementary Figure 3. The socioecological model (SEM) applied to malaria interventions at multiple levels

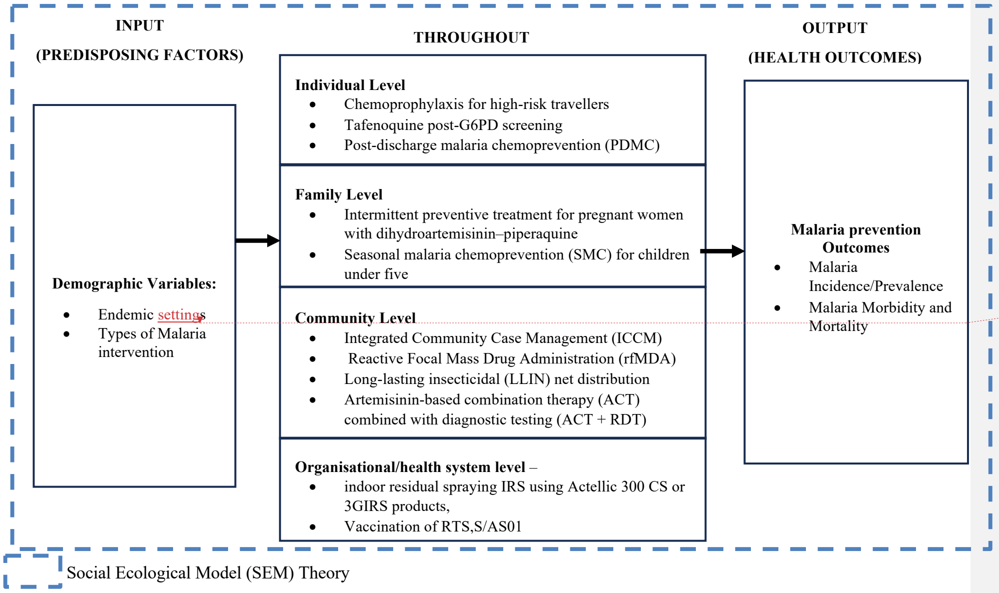

Deleted: countrie
