# Supplementary material for: Strategic resource allocation for malaria elimination in endemic settings: a systematic review of cost-effectiveness evidence
Source: Front Public Health. 2026 Feb 9;13:1718225. doi: 10.3389/fpubh.2025.1718225 (PMC12926903; doi:10.3389/fpubh.2025.1718225)
Supplement: Supplementary file 2 [file Data_Sheet_2.pdf]

Supplementary Material

1 Supplementary Tables

1.1 Supplementary Tables

1.1.1 Table 1. Comparison of Identified PROSPERO and Cochrane Records Related to Malaria Interventions

No	Database	ID / Link	Title	Year	Focus / Notes	Uniqueness vs Our Review
**1**	PROSPERO	CRD42023436966	Optimisation models for malaria programming: a systematic review	2023	Optimization models	Not focused on cost-effectiveness or SEM policy modeling
**2**	PROSPERO	CRD42018105625	Systematic review on economic evaluations of malaria interventions	2018	Economic evaluations	Lacks updated 2018–2025 evidence and SEM framework
**3**	PROSPERO	CRD42025633693	Economic evaluations for malaria vaccines: A systematic review	2025	Vaccine interventions	Limited to vaccines, not all malaria interventions
**4**	PROSPERO	CRD42018102294	Systematic review on economic evaluations of malaria interventions	2018	Economic evaluations	Does not integrate SEM-based policy modeling across multi-endemic contexts
**5**	Cochrane	10.1002/14651858.CD009186.pub2	Strategies to increase ownership and use of insecticide-treated bednets to prevent malaria	ongoing	Behavioral interventions	Not cost-effectiveness or resource allocation focus
**6**	Cochrane	10.1002/14651858.CD006404.pub5	Pyronaridine-artesunate for treating uncomplicated Plasmodiumfalciparum malaria	ongoing	Clinical efficacy	Not cost-effectiveness or SEM-based policy
**7**	Cochrane	10.1002/14651858.CD008923.pub3	Mosquito aquatic habitat modification and	ongoing	Vector control	Not cost-effectiveness or SEM-based policy

			manipulation interventions to control malaria			
**8**	Cochrane	10.1002/14651858.CD006491.pub4	Mefloquine for preventing malaria during travel to endemic areas	ongoing	Clinical prevention	Not cost-effectiveness or SEM-based policy
**9**	Cochrane	10.1002/14651858.CD000386.pub2	Chloroquine or amodiaquine plus sulfadoxine-pyrimethamine for treating uncomplicated malaria	ongoing	Clinical efficacy	Not cost-effectiveness or SEM-based policy
**10**	Cochrane	10.1002/14651858.CD009926.pub2	Subsidising artemisinin-based combination therapy in the private retail sector	ongoing	Policy interventio n	Not integrated SEM-based resource allocation
**11**	Cochrane	10.1002/14651858.CD012504.pub2	Interventions for investigating and identifying the causes of stillbirth	ongoing	Not malaria-related	Not related to cost-effectiveness or malaria resource allocation
**12**	Cochrane	10.1002/14651858.CD004966.pub2	Sulfadoxine-pyrimethamine plus artesunate vs sulfadoxine-pyrimethamine plus amodiaquine for uncomplicated malaria	ongoing	Clinical efficacy	Not cost-effectiveness or SEM policy modeling
**13**	Cochrane	10.1002/14651858.CD008122.pub2	Rapid diagnostic tests for diagnosing uncomplicated P. falciparum malaria in endemic countries	ongoing	Diagnostic s	Not cost-effectiveness or SEM policy modeling
**14**	Cochrane	10.1002/14651858.CD008145.pub4	Interventions for improving coverage of childhood immunisation in low- and middle-income countries	ongoing	Immunizat ion	Not malaria-specific cost-effectiveness
**15**	Cochrane	10.1002/14651858.CD009604.pub2	Fortification of condiments and seasonings with iron for preventing anaemia and improving health	ongoing	Nutrition	Not malaria-related

1.1.2 Table 2. The keywords used to search related articles in the systematic review

Database	Search String
**Scopus**	(((((("cost-effectiveness analysis") OR ("cost analysis")) OR ("cost benefit analysis")) OR ("cost utility analysis")) OR ("cost minimization analysis")) OR ("economic evaluation")) AND (((((malaria) OR ("human malaria")) OR ("zoonotic malaria")) OR ("plasmodium malaria")) OR ("Protozoa malaria")))
**Web of Science**	((((((TI=(cost-effectiveness analysis)) OR TI=(cost analysis)) OR TI=(cost benefit analysis)) OR TI=(cost utility analysis)) OR TI= (cost minimization analysis)) OR TI= (economic evaluation)) AND (((((TI=malaria)) OR TI= (human malaria)) OR TI= (zoonotic malaria)) OR TI=(plasmodium malaria)) OR TI=(Protozoa malaria)
**PubMed**	((((((cost-effectiveness analysis [Title/Abstract] OR (cost analysis[Title/Abstract])) OR (cost benefit analysis[Title/Abstract])) OR (cost utility analysis[Title/Abstract])) OR (cost minimization analysis[Title/Abstract])) OR (economic evaluation[Title/Abstract])) AND (((((malaria[Title/Abstract]) OR (human malaria[Title/Abstract])) OR (zoonotic malaria[Title/Abstract])) OR (plasmodium malaria[Title/Abstract])) OR (Protozoa malaria[Title])))

1.1.3 Table 3. Joanna Briggs Institute's Critical Appraisal Tools

Article No	Author (year)	Study Design	MMAT Criteria (Y/N/CT)	Overall Appraisal	Comment
**1.**	Arroz et al (2019)	Cross -sectional	8/8 score Y	100% (High)	High quality, included in synthesis
**2.**	Lubogo et al (2021)	Cross -sectional	8/8 score Y	100% (High)	High quality, included in synthesis
**3.**	Hailu et al (2018)	Randomized Controlled Trial (RCT)	12/13 score Y	92% (High)	Strong methodology, included
**4.**	Kuhl et al (2019)	Randomized Controlled Trial (RCT)	12/13 score Y	92% (High)	Strong methodology, included
**5.**	Sarker & Sultana (2020)	Randomized Controlled Trial (RCT)	12/13 score Y	92% (High)	Strong methodology, included
**6.**	Du et al (2020)	Randomized Controlled Trial (RCT)	12/13 score Y	92% (High)	Strong methodology, included
**7.**	Fernandes et al (2020)	Randomized Controlled Trial (RCT)	12/13 score Y	92% (High)	Strong methodology, included

**8.**	Paintain et al (2020)	Randomized Controlled Trial (RCT)	12/13 score Y	92% (High)	Strong methodology, included
**9.**	Shepard et al (2020)	Randomized Controlled Trial (RCT)	12/13 score Y	92% (High)	Strong methodology, included
**10.**	Alonso et al (2021)	Randomized Controlled Trial (RCT)	12/13 score Y	92% (High)	Strong methodology, included
**11.**	Gilmartin et al (2021)	Randomized Controlled Trial (RCT)	12/13 score Y	92% (High)	Strong methodology, included
**12.**	Avancena et al (2022)	Randomized Controlled Trial (RCT)	12/13 score Y	92% (High)	Strong methodology, included
**13.**	Ntuku et al (2022)	Randomized Controlled Trial (RCT)	12/13 score Y	92% (High)	Strong methodology, included
**14.**	Kühl et al (2022)	Randomized Controlled Trial (RCT)	12/13 score Y	92% (High)	Strong methodology, included
**15.**	Yukich et al (2022)	Randomized Controlled Trial (RCT)	12/13 score Y	92% (High)	Strong methodology, included
**16.**	Brito-Sousa et al (2022)	Randomized Controlled Trial (RCT)	12/13 score Y	92% (High)	Strong methodology, included
**17.**	Fiorina et al (2022)	Randomized Controlled Trial (RCT)	12/13 score Y	92% (High)	Strong methodology, included
**18.**	Price et al (2024)	Randomized Controlled Trial (RCT)	12/13 score Y	92% (High)	Strong methodology, included

Y=yes, N=No, U=Unclear, NA= Not applicable, **Q1–Q8 (Cross-sectional):** clarity of inclusion criteria, description of study population and setting, validity of measurement, identification of confounders, strategies to deal with confounders, appropriateness of statistical analysis, etc, **Q1–Q13 (RCTs):** sequence generation, allocation concealment, blinding, similarity of groups, completeness of follow-up, validity of outcome measures, appropriateness of analysis, etc**; > 90% = High quality; 80–90% = Moderate; <80% = Low**

1.1.4 Table 4. The characteristics of all the studies are included in the systematic review

No.	Author, Year	Country	Study Design	Study Period	Population	Intervention category	Costing perspective	Malaria Intervention: Intervention	Malaria Intervention: Comparator	Economic outcome
1	**Hailu et al (2018)**	Africa	RCT	2014 - 2016	General population	Preventive measures	Provider	Combined LLIN+IRS	LLIN and IRS alone	DALYs averted
2	**Kuhl et al (2019)**	Africa	RCT	2015	Children		Provider & Societal	SMC	Health promotion	Case Averted; Death Averted
3	**Arroz et al (2019)**	Africa	Cross-sectional	2015	General population		Provider	LLIN (mode delivery)	LLIN standard delivery	HH per bed net universal coverage (one LLIN for every two persons)
4	**Shepard et al (2020)**	Africa	RCT	2010 - 2014	General population		Provider	LLINs with a synergist, PBO	Standard LLIN	Case Averted; DALYs averted
5	**Alonso et al (2021)**	Africa	RCT	2016 - 2018	Children		Provider	IRS with pirimiphos-methyl (Actellic 300 CS)	ITN	Case Averted; Death averted; DALYs averted
6	**Gilmartin et al (2021)**	Africa	RCT	2016	Children		Provider	SMC	NR	Case Averted; Death averted
7	**Kühl et al (2022)**	Africa	RCT	2016 - 2018	Children		Provider & Societal	PDMC	NR	QALY gained
8	**Yukich et al (2022)**	Africa	RCT	2016 - 2019	General population		Provider	IRS programmes using 3GIRS products	Standard IRS	Case Averted; Death averted; DALYs averted
9	**Fiorina et al (2022)**	Paris	RCT	2018	Traveller		Provider	Drugs for malaria CP	NR	Case Averted
10	**Sarker & Sultana et al (2020)**	India	RCT	2019	Children	Treatment measure	Provider & Societal	Childhood malaria vaccination type RTS,S/AS01	NR	Case averted; Death Averted; DALYs averted
11	**Fernandes et al (2020)**	Africa	RCT	2019	Pregnant women		Provider	IPT with dihydroartemisinin–piperaquine	No intervention	DALYs averted of Maternal and Child

12	**Paintain et al (2020)**	Africa	RCT	2013 - 2016	Pregnant women		Provider	IPT with screening	Screening IPT alone	DALYs averted of Maternal and Child
13	**Lubogo rt al (2021)**	Africa	Quasi experimental & cross-sectional	2013 - 2015	General population		Provider	ICCM drug seller training	ICCM without drug seller training	Case Averted
14	**Avancena et al (2022)**	Africa	RCT	2020	Children		Provider	Combination of Treatment ACT and diagnostic RDT	Diagnostic RDT alone	DALYs averted; QALY gained
15	**Ntuku et al (2022)**	Africa	RCT	2017	General population		Provider	rfMDA	RACD	Case Averted; DALYs Averted
16	**Du et al (2020)**	China	RCT	2018 - 2019.	General population	Diagnostic measure	Provider	RDT with microscopic	RDT and microcopy alone.	Case averted
17	**Brito-Sousa et al (2022)**	Brazil	RCT	2020	General population		Provider	G6PD screening	NR	PQ associated hospitalization avoided
18	**Price et al (2024)**	Brazil	RCT	2020 - 2021	General population		Provider	Tafenoquine after G6PD screening	Primaquine without G6PD screening (standard)	DALYs averted

Abbreviations: NR, not reported; RCT, randomised controlled trial; LLIN, long-lasting insecticidal net; IRS, indoor residual spraying; G6PD, glucose-6-phosphate dehydrogenase; RACD, reactive case detection; ICCM, integrated community management; IPT, intermittent preventive treatment; CP, chemoprophylaxis; PDMC, post-discharge malaria chemoprevention; PBO, piperonyl butoxide; ACT, artemisinin-based combination therapy; HH, household; PQ, primaquine.

1.1.5 Table 5. Synthesis of Cost-Effectiveness and Policy Recommendation for Malaria Intervention

Intervention Category	Specific Intervention	No. of Supporting Studies	ICER Range (2024 USD/DALY) *	Dominance Status	Cost-Effectiveness Classification	Policy Implication & Recommendation
**Preventive (Vector Control)**	LLINs (incl. PBO nets)	3 (Africa)	Highly cost-effective	Cost-Effective	Highly Cost-Effective	A foundational intervention. Prioritize universal coverage and deploy PBO nets in resistance areas.
	IRS (incl. Actellic, 3GIRS)	3 (Africa)	USD18 – USD75	Cost-Effective	Highly Cost-Effective	A high-priority investment for high-transmission areas. Combine with LLINs where feasible.
	LLINs + IRS (Combined)	1 (Africa)	USD 22 – USD 85	Cost-Effective	Highly Cost-Effective	**Highest impact.** Recommended as a primary strategy in high-transmission/resistance areas.
**Preventive (Chemoprevention)**	SMC / PDMC	3 (Africa)	USD 8 – USD 28	Often Dominant	Highly Cost-Effective	**Top priority for children <5** in seasonal zones. Should be scaled up.
**Treatment**	IPT (Pregnancy)	2 (Africa)	USD 15 – USD 65	Often Dominant	Highly Cost-Effective	**Integrate into routine ANC.** Essential for improving maternal and child survival.
	ACT + RDT	1 (Africa)	USD 25 – USD 95	Often Dominant	Highly Cost-Effective	**Standard practice.** Maintain and strengthen to ensure rational treatment and combat resistance.
	Vaccination (RTS,S)	1 (India)	USD 45 – USD 142	Cost-Effective	Cost-Effective to Highly Cost-Effective	A **complementary tool.** Introduce alongside existing interventions, considering long-term funding.
**Diagnostics & Special Cases**	RDT + Microscopy	1 (China)	Cost-effective	Cost-Effective	Cost-Effective	Supports rational use of drugs. Continue deployment as part of diagnostic-led strategies.
	G6PD Screening + Tafenoquine	2 (Brazil)	USD 75 – USD 285	Not Dominant	Context-Specific	**Critical for *P. vivax* elimination.** Pilot and scale where screening is affordable and feasible.
	Chemoprophylaxis (Travellers)	1 (France)	Cost-effective	Cost-Effective	Cost-Effective (for target group)	Relevant for **travel medicine** in non-endemic countries; not a primary strategy for endemic regions.

Abbreviations: ANC, Antenatal care; DALY, disability-adjusted life year; QALY, quality-adjusted life year; HH, household; PBO, piperonyl butoxide; RACD, reactive case detection; ACT, artemisinin-based combination therapy; CP, chemoprophylaxis; G6PD, glucose-6-phosphate dehydrogenase deficiency; NR, not reported. *All monetary values were adjusted to 2024 USD using the US Consumer Price Index (CPI); **ICER**, Incremental Cost-Effectiveness Ratio. Ranges are based on 2024 USD. WHO cost-effectiveness thresholds were applied. **Dominance Status:** Indicates if the intervention is more effective and less costly (**Dominant**) than the comparator, or simply a good value (**Cost-Effective**). **Cost-Effectiveness Classification: Highly Cost-Effective:** ICER < GDP per capita; **Cost-Effective:** ICER between 1-3x GDP per capita; **Context-Specific:** Economic outcome depends heavily on local factors like price and capacity.

Deleted: effectiveness
Formatted Table
Deleted: $
Deleted: $
Deleted: $
Deleted: $
Deleted: $
Deleted:
Deleted: $
Deleted: $
Deleted: $
Deleted: $
Deleted: $
Deleted: $
Deleted: $
Deleted: $
Deleted: $
Deleted: Cost per DALY averted.
Formatted: Font: 9 pt

1.1.6 Table 6 PRISMA 2020 Checklist

Section and Topic	Item #	Checklist item	Reported on page #
**TITLE**			
Title	1	Identify the report as a systematic review.	1
**ABSTRACT**			
Abstract	2	See the PRISMA 2020 for Abstracts checklist.	16
**INTRODUCTION**			
Rationale	3	Describe the rationale for the review in the context of existing knowledge.	2
Objectives	4	Provide an explicit statement of the objective(s) or question(s) the review addresses.	3
**METHODS**			
Eligibility criteria	5	Specify the inclusion and exclusion criteria for the review and how studies were grouped for the syntheses.	4
Information sources	6	Specify all databases, registers, websites, organisations, reference lists and other sources searched or consulted to identify studies. Specify the date when each source was last searched or consulted.	3
Search strategy	7	Present the full search strategies for all databases, registers and websites, including any filters and limits used.	3 &14
Selection process	8	Specify the methods used to decide whether a study met the inclusion criteria of the review, including how many reviewers screened each record and each report retrieved, whether they worked independently, and if applicable, details of automation tools used in the process.	4-5
Data collection process	9	Specify the methods used to collect data from reports, including how many reviewers collected data from each report, whether they worked independently, any processes for obtaining or confirming data from study investigators, and if applicable, details of automation tools used in the process.	4
Data items	10a	List and define all outcomes for which data were sought. Specify whether all results that were compatible with each outcome domain in each study were sought (e.g. for all measures, time points, analyses), and if not, the methods used to decide which results to collect.	4 & 6
	10b	List and define all other variables for which data were sought (e.g. participant and intervention characteristics, funding sources). Describe any assumptions made about any missing or unclear information.	4
Study risk of bias assessment	11	Specify the methods used to assess risk of bias in the included studies, including details of the tool(s) used, how many reviewers assessed each study and whether they worked independently, and if applicable, details of automation tools used in the process.	5
Effect measures	12	Specify for each outcome the effect measure(s) (e.g. risk ratio, mean difference) used in the synthesis or presentation of results.	3&6
Synthesis methods	13a	Describe the processes used to decide which studies were eligible for each synthesis (e.g. tabulating the study intervention characteristics and comparing against the planned groups for each synthesis (item #5)).	4&5

Section and Topic	Item #	Checklist item	Reported on page #
	13b	Describe any methods required to prepare the data for presentation or synthesis, such as handling of missing summary statistics, or data conversions.	4
	13c	Describe any methods used to tabulate or visually display results of individual studies and syntheses.	6-8
	13d	Describe any methods used to synthesize results and provide a rationale for the choice(s). If meta-analysis was performed, describe the model(s), method(s) to identify the presence and extent of statistical heterogeneity, and software package(s) used.	4
	13e	Describe any methods used to explore possible causes of heterogeneity among study results (e.g. subgroup analysis, meta-regression).	-
	13f	Describe any sensitivity analyses conducted to assess robustness of the synthesized results.	-
Reporting bias assessment	14	Describe any methods used to assess risk of bias due to missing results in a synthesis (arising from reporting biases).	-
Certainty assessment	15	Describe any methods used to assess certainty (or confidence) in the body of evidence for an outcome.	5, 7-8
**RESULTS**			
Study selection	16a	Describe the results of the search and selection process, from the number of records identified in the search to the number of studies included in the review, ideally using a flow diagram.	4&5
	16b	Cite studies that might appear to meet the inclusion criteria, but which were excluded, and explain why they were excluded.	-
Study characteristics	17	Cite each included study and present its characteristics.	5&6
Risk of bias in studies	18	Present assessments of risk of bias for each included study.	5
Results of individual studies	19	For all outcomes, present, for each study: (a) summary statistics for each group (where appropriate) and (b) an effect estimate and its precision (e.g. confidence/credible interval), ideally using structured tables or plots.	6-8
Results of syntheses	20a	For each synthesis, briefly summarise the characteristics and risk of bias among contributing studies.	5-8
	20b	Present results of all statistical syntheses conducted. If meta-analysis was done, present for each the summary estimate and its precision (e.g. confidence/credible interval) and measures of statistical heterogeneity. If comparing groups, describe the direction of the effect.	6-8
	20c	Present results of all investigations of possible causes of heterogeneity among study results.	-
	20d	Present results of all sensitivity analyses conducted to assess the robustness of the synthesized results.	-
Reporting biases	21	Present assessments of risk of bias due to missing results (arising from reporting biases) for each synthesis assessed.	-
Certainty of evidence	22	Present assessments of certainty (or confidence) in the body of evidence for each outcome assessed.	7-8
**DISCUSSION**			

Section and Topic	Item #	Checklist item	Reported on page #
Discussion	23a	Provide a general interpretation of the results in the context of other evidence.	4
	23b	Discuss any limitations of the evidence included in the review.	8-9
	23c	Discuss any limitations of the review processes used.	10
	23d	Discuss implications of the results for practice, policy, and future research.	8-10
**OTHER INFORMATION**			
Registration and protocol	24a	Provide registration information for the review, including register name and registration number, or state that the review was not registered.	-
	24b	Indicate where the review protocol can be accessed, or state that a protocol was not prepared.	-
	24c	Describe and explain any amendments to information provided at registration or in the protocol.	-
Support	25	Describe sources of financial or non-financial support for the review, and the role of the funders or sponsors in the review.	11
Competing interests	26	Declare any competing interests of review authors.	10
Availability of data, code and other materials	27	Report which of the following are publicly available and where they can be found: template data collection forms; data extracted from included studies; data used for all analyses; analytic code; any other materials used in the review.	Data is contained in the article

1.1.7 Table 7 PRISMA 2020 Abstract Checklist

Section and Topic	Item #	Checklist item	Reported (Yes/No)
**TITLE**			
Title	1	Identify the report as a systematic review.	Yes
**BACKGROUND**			
Objectives	2	Provide an explicit statement of the main objective(s) or question(s) the review addresses.	Yes
**METHODS**			
Eligibility criteria	3	Specify the inclusion and exclusion criteria for the review.	Yes
Information sources	4	Specify the information sources (e.g. databases, registers) used to identify studies and the date when each was last searched.	Yes
Risk of bias	5	Specify the methods used to assess risk of bias in the included studies.	Yes

Section and Topic	Item #	Checklist item	Reported (Yes/No)
Synthesis of results	6	Specify the methods used to present and synthesise results.	Yes
**RESULTS**			
Included studies	7	Give the total number of included studies and participants and summarise relevant characteristics of studies.	Yes
Synthesis of results	8	Present results for main outcomes, preferably indicating the number of included studies and participants for each. If meta-analysis was done, report the summary estimate and confidence/credible interval. If comparing groups, indicate the direction of the effect (i.e. which group is favoured).	Yes
**DISCUSSION**			
Limitations of evidence	9	Provide a brief summary of the limitations of the evidence included in the review (e.g. study risk of bias, inconsistency and imprecision).	Yes
Interpretation	10	Provide a general interpretation of the results and important implications.	Yes
**OTHER**			
Funding	11	Specify the primary source of funding for the review.	Yes
Registration	12	Provide the register name and registration number.	No
